# Structural basis for broad neutralization of rabies virus by an antibody cocktail SYN023

**DOI:** 10.1080/22221751.2025.2547724

**Published:** 2025-08-19

**Authors:** Lei Cao, Jiagui Qu, Chuziyue Zhang, Ruihong Chen, Jinyue Wang, Christine Fehlner-Gardiner, Michael Niezgoda, Panayampalli Subbian Satheshkumar, Xiangxi Wang, Eric Tsao

**Affiliations:** a National Laboratory of Macromolecules, Institute of Biophysics, Chinese Academy of Sciences, Beijing, People’s Republic of China; b Synermore Biologics, Suzhou, People’s Republic of China; c University of Chinese Academy of Sciences, Beijing, People’s Republic of China; d Centre of Expertise for Rabies, Ottawa Laboratory–Fallowfield, Canadian Food Inspection Agency, Ottawa, Canada; e Poxvirus and Rabies Branch, Division of High–Consequence Pathogens and Pathology, National Center for Emerging and Zoonotic Infectious Diseases, CDC, Atlanta, USA

## Dear Editor,

Rabies is a severe zoonotic disease with nearly 100% mortality once symptoms appear [1,2]. Prevention relies on prompt post-exposure prophylaxis (PEP), which includes vaccination and the administration of anti-rabies immunoglobulin (RIG) for immediate immune protection [3]. However, RIG use is constrained by supply, cost, and safety concerns [4]. The World Health Organization (WHO) recommends monoclonal antibodies (mAbs) as safer alternatives [5]. We revisited SYN023, a monoclonal antibody cocktail comprising two humanized antibodies: CTB011, which targets antigenic site III, and CTB012, binding an undefined region near loop 4 [6], reported in the 2017 in order to study the mechanism of neutralization of rabies virus (RABV) by these antibodies at the atomic level. These antibodies neutralize RABV isolates from China and North America, demonstrating protective efficacy in PEP studies with animals [7]. Despite its approval in China in 2024, SYN023 remains under investigation to clarify its antigen recognition and neutralization mechanisms [8]. In this study, we systematically analysed the immunological properties of these two monoclonal antibodies and elucidated the structural basis of antibody-mediated RABV neutralization.

**Figure 1.**
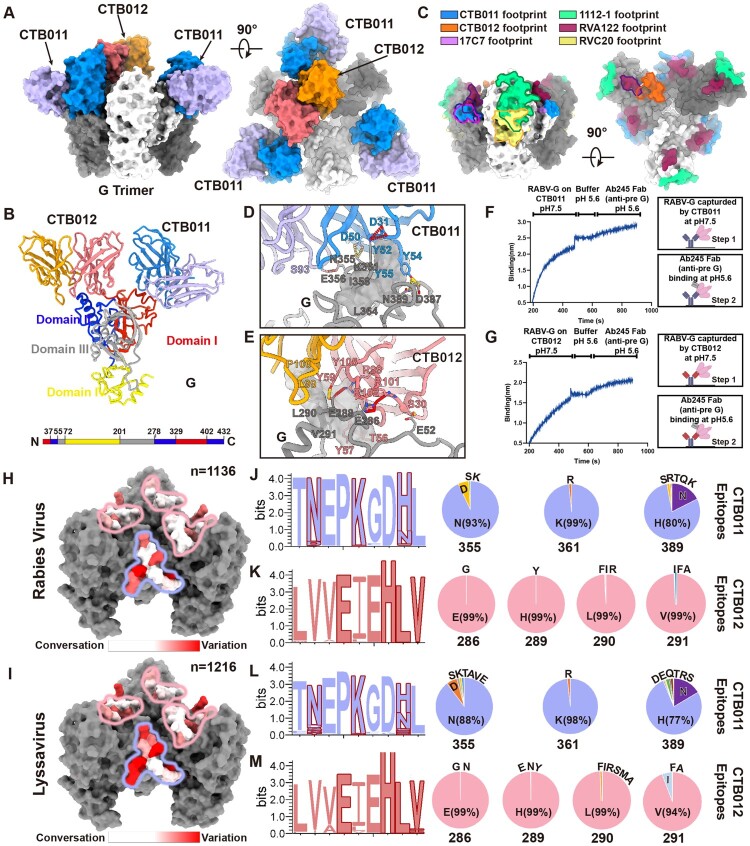
Structure of broadly neutralizing antibody CTB011 and CTB012. (A) Orthogonal views of the CTB011-CTB012-RABV G complex. (B) The variable regions of the Fab fragments of mAbs CTB011 and CTB012 bound to a single RABV-G protomer are shown as ribbons. (C) Structurally characterized anti-RABV antibody epitopes, with footprints of various antibodies mapped on trimeric RABV-G. (D) Interaction between CTB011 (heavy chain: blue; light chain: purple) and RABV-G (grey). (E) Interaction between CTB012 (heavy chain: red; light chain: orange) and RABV-G (grey). (F) Locking of RABV-G in pre-fusion conformation by CTB011. (G) Locking of RABV-G in pre-fusion conformation by CTB012. (H, I) The conservation in CTB011/CTB012 epitopes across 1134 RABV and 1,216 lyssavirus, shown as surface maps, the CTB011 and CTB012 epitopes were heighted by bule and red circles, respectively. (J–M) Amino acid residue conservation in CTB011/CTB012 epitopes across 1134 RABV and 1216 lyssavirus (left), the key contact residues were coloured by red. Frequency of amino acid residues in CTB011/CTB012 epitopes (right).

To elucidate the molecular basis of RABV neutralization by CTB011 and CTB012, we employed single-particle cryo-electron microscopy (cryo-EM) to resolve the structure of trimeric RABV-G in complex with the antigen-binding fragments (Fabs) of CTB011 and CTB012 at a resolution of 3.9 Å (Figure 1(A); Figure S1 and S2; Table S1). This analysis revealed that Fab CTB011 binds to each protomer of the RABV-G trimer within domain I (Figure 1(A,B)). In contrast, Fab CTB012 binds exclusively to a single G monomer at the apex of domain II due to steric hindrance (Figure 1(A,B), S3). Neutralizing antibodies targeting the rabies virus glycoprotein (RABV-G) have been characterized through four distinct structural complexes. Among these, mAbs 17C7 and RVA122 bind to epitopes within domains I and II, while mAbs RVC20 and 1112-1 interact with domain III [9,10]. The binding region of CTB011 constitutes approximately 620 Å^2^ of buried surface area within the previously identified Site III in Domain I, exhibiting substantial overlap with that of mAb 17C7 and partial overlap with mAb RVA122 (Figure 1(C)). Whereas CTB012 binds to a novel, uncharacterized epitope predominantly formed by residues in loop 4 of domain II, with its Fab region burying approximately 600 Å^2^ (Figure 1(C)). In both the antibody-bound RABV- G structures, the heavy chain and light chain variable domains contribute ∼90% and ∼10% of the protein–protein interface, respectively.

CTB011's paratope consists of four complementarity-determining regions (CDRs): CDR L3 of the light chain and all three heavy chain CDRs (H1, H2, H3). Tight binding is facilitated by extensive hydrophobic interactions between specific residues (W354, I357, I358, L364, V390) on Domain I and CDR residues on the heavy chain (Y52, Y54, P53, Y55), as well as hydrophilic interactions involving Domain I residues (R352, N355, E356, K361, N387, N389) and heavy chain residues (D31, D50, Y54, S93) (Figure 1(D)). Notably, residues N355, K361, and N389 play critical roles in CTB011 binding, as supported by prior mutagenesis studies [6]. CTB012, in contrast, engages a distinct epitope at the N-terminus of the central helix and Loop 4. Hydrophobic interactions, involving residues L47, V48, V49, E286, H289, L290, and V291 on Domain I, alongside complementary heavy and light chain residues (T50, Y57, T58, Y59, L99, P100), are key to tight binding (Figure 1(E)). Additionally, hydrophilic interactions between Domain I residues (E52, E286, E288) and heavy chain residues (S30, Y59, R99, R101, R102) further stabilize the interaction (Figure 1(E)).

We next investigated the neutralizing mechanisms of CTB011 and CTB012. In our structural, Fab CTB011 forms hydrogen bonds with Arg352, a key residue involved the p75 neurotrophin receptor (p75NTR) recognition site, potentially disrupting RABV's neuroinvasion pathway [11]. However, as p75NTR is not essential for pathogenicity [12], suggesting that receptor blockade is unlikely to be CTB011's primary mechanism. An alternative neutralization mechanism involves inhibiting the conformational transition of RABV-G from the prefusion to postfusion state, which drives membrane fusion required for viral genome delivery into the host cell cytoplasm. To analyse CTB011 binding in the postfusion context, we generated a trimeric postfusion RABV-G model by integrating the trimeric VSV-G postfusion structure with the low-pH-derived RABV monomeric G^ecto^ structure [13] (Figure S4). Superimposing the CTB011 Fab-RABV-G domain III structure onto this model revealed minimal conformational changes in domain III. Nevertheless, these changes resulted in steric clashes between bound CTB011 Fab and postfusion RABV-G, suggesting that CTB011 binding may inhibit the structural rearrangement required for membrane fusion (Figure S4B). The epitope recognized by CTB012 Fab localizes to loop 4, a structurally critical region previously shown to maintain the prefusion architecture and mediate its transition to the postfusion conformation [9]. By binding to this region, CTB012 blocks the conformational change, thereby constituting its likely primary neutralization mechanism (Figure S4C). To further assess the impact of CTB011 and CTB012 on pH-induced conformational changes in RABV-G, we employed a “sandwich-configuration” BLI assay. When RABV-G was captured by CTB011 or CTB012 under neutral conditions and exposed to pH 5.6 for 300 s, mAb Ab245, which has been demonstrated to recognize only the pre-fusion conformation (Figure S5A, B), maintained binding capacity even at acidic pH (Figure 1(F,G), Figure S5C). These findings indicate that pre-binding of RABV-G by CTB011 or CTB012 in the pH-neutral (prefusion) conformation prevents the pH-triggered conformational change required for Ab245 binding.

To predict the broad neutralization potential of CTB011 and CTB012, we analysed epitope conservation across 1216 Lyssavirus sequences, including 1136 RABV isolates from the UniProtKB database (Figure 1(H,I)). CTB012's epitope was highly conserved across both RABV and Lyssavirus species, supporting its broad reactivity. In contrast, CTB011's epitope showed less conservation, though key contact residues remained consistent. However, the key contact residues of the CTB011 and CTB012 mAbs [6,14] are highly conserved across RABV isolates and Lyssavirus (Figure 1(H–M)).

The antigenic site targeted by the CTB011 monoclonal antibody exhibits some variability, notably at position 389, which is polymorphic (Figure 1(J,L)). Approximately 80% of street strains possess histidine at this position. Our prior studies have shown that the neutralizing data confirm that histidine substitution at N389 does not impact CTB011's neutralization capacity [6], as it does not alter interactions with antibody CDR H2 but rather stabilizes nearby π-stacking. Another polymorphic site, position 355, has asparagine in 93% of strains; the rest carry aspartic acid, serine, or lysine, which may evade CTB011 neutralization due to steric or interaction disruptions. The position 361 predominantly features lysine (98.6%), while only 1.4% have arginine. Previous neutralization tests demonstrated that amino acid substitutions at this site do not affect CTB011's efficacy [14] due to a highly conserved interaction between G and acidic residues on CDR H1.

Analysis of the CTB012 target epitope, spanning residues 286–291 of the RABV G protein, revealed high conservation of key residues 286 and 289 (>99.8%), while positions 290 and 291 are polymorphic (Figure 1(K,M)). Notably, substitutions with large side-chain amino acids like phenylalanine induce steric hindrance, preventing CTB012 binding [6]. Despite this, site 291 mostly features small hydrophobic residues (>99.9%), maintaining neutralization potential. Thus, the conserved epitopes of CTB012 complement CTB011's limitations, enhancing the broad-spectrum activity of the SYN023 antibody cocktail. The breadth of SYN023's neutralizing ability was also evaluated accross 28 RABV isolates representing major circulating lineages (RAC-SK, Cosmopolitan, Arctic-related and Bat), as well as 4 non-RABV phylogroup I isolates. SYN023 demonstrated potent neutralization against all tested RABV and non-RABV isolates (Table S4) further confirming its broad neutralizing spectrum.

## Discussion

This study elucidates the neutralization mechanisms and broad-spectrum efficacy potential of SYN023, a monoclonal antibody cocktail designed to target RABV and related Lyssaviruses. The findings reveal the complementary actions of its two components, CTB011 and CTB012. CTB011 binds to antigenic site III within Domain I, while CTB012 targets a novel epitope near Loop 4, which bridges Domains II and III. Structural analysis using cryo-electron microscopy uncovered their distinct binding modes on the trimeric RABV-G protein, demonstrating how these antibodies stabilize the glycoprotein in its pre-fusion conformation, thereby inhibiting viral entry. Notably, CTB012 exhibits superior neutralization of non-RABV Lyssaviruses compared to CTB011, enhancing the overall efficacy of SYN023 against diverse viral lineages.

Currently, over 20 types of equine or human plasma-derived RIG are available for PEP after rabies virus exposure. Annually, globally, 20 million individuals exposed to RABV require PEP, yet fewer than 2% of those with category III exposures receive RIG. Low RIG adoption is due to cost, safety, and supply issues. To address this, the WHO strongly advocates for antibody cocktail as practical RIG alternatives, especially in less developed countries. The ideal characteristics of rabies virus-neutralizing antibody mixtures include high neutralizing potency, recognition of distinct non-overlapping antigenic epitopes, and broad neutralizing activity against wild-type RABV isolates to achieve full coverage and prevent escape. In previous studies, SYN023 not only demonstrated the same breadth of neutralization as HRIG but also showed significantly higher efficacy against half of the North American wildlife RABV strains [6].

The findings have substantial implications for rabies prevention and global health. SYN023 provides a safer, more reliable alternative to RIG, effectively mitigating issues such as supply shortages and safety concerns associated with human- or equine-derived products. Its broad-spectrum efficacy is particularly valuable in regions where the genetic diversity of RABV undermines conventional therapies. Moreover, the structural insights gained from this study can inform the design of next-generation monoclonal antibodies or antibody cocktails with enhanced efficacy against Lyssaviruses.

## Supplementary Material

Supplementary information0615.docx

## Data and materials availability

All data associated with this study are present in the paper or the Supplementary Materials and can also be obtained from the corresponding author upon request. The cryo-EM maps and atomic models have been deposited at the Electron Microscopy Data Bank and the Protein Data Bank with accession codes EMD-63979, and PDB: 9UA5 (RABV G binding to CTB011 and CTB012 Fab).
