# Supplementary material for: Structural basis for broad neutralization of rabies virus by an antibody cocktail SYN023
Source: Emerg Microbes Infect. 2025 Aug 19;14(1):2547724. doi: 10.1080/22221751.2025.2547724 (PMC12667334; doi:10.1080/22221751.2025.2547724)
Supplement: Supplementary information0615.docx [file TEMI_A_2547724_SM9407.docx]

**Supplementary information**

**Methods**

**Protein expression and purification**

The codon-optimized sequences for the RABV-G protein (PV strain) with a C-terminal Strep II tag were expressed in 293F cells. Protein purification was initiated by treating thawed pellets with an extraction buffer (50 mM HEPES, pH 7.5, 150 mM NaCl, 1% n-octyl-β-d-glucoside [Generon], cOmplete Protease Inhibitor cocktail [Roche]) for 2 hours at 4°C. The lysate was clarified by centrifugation at 13,000 × g for 30 minutes. RABV-G protein then purified from the clarified lysate using a Strep-Tactin XT Superflow column (IBA Lifesciences) following the manufacturer’s protocol, with the wash and elution buffers supplemented with 1% n-octyl-β-d-glucoside. Fractions containing the purified protein complex were mixed with a three-fold mass excess of A8-35 amphipol (Anatrace) and incubated at 4 °C for 1 hour. Detergent removal was performed using Pierce detergent removal spin columns (ThermoFisher Scientific), and the sample was concentrated using an Amicon centrifugal concentrator (Millipore). The CTB011 and CTB012 Fab were added to saturate available binding sites, and the resulting RABV-G-CTB011-CTB012 Fab complex was further purified by size-exclusion chromatography on a Superose 6 10/300 Increase column (Cytiva) in a buffer containing 50 mM HEPES, pH 7.5, and 150 mM NaCl.

**Cryo-EM sample preparation and data collection**

Purified protein was concentrated to 1.2 mg/ml for sample preparation. Holy-carbon gold grid (Cflat R1.2/1.3 mesh 300) was freshly glow-discharged with a Solarus 950 plasma cleaner (Gatan) for the 20 s. A 3 μL aliquot of the mixture complex was transferred onto the grids, blotted with filter paper at 22°C and 100% humidity, and plunged into the ethane using a Vitrobot Mark IV (FEI). For the complex, micrographs were collected at 300 kV using a Titan Krios microscope (ThermoFisher), equipped with a K3 detector (Gatan, Pleasanton, CA), using SerialEM automated data collection software movies (32 frames, every 0.2 s, total dose 60 e-Å-2) were recorded at a final pixel size of 1.07 Å with a defocus of between -1.5 and -2.0 μm.

**Image processing**

For the RABV-G-CTB011-CTB012 Fab complex, a total of 3,859 micrographs were recorded, respectively. Almost equivalent image processing strategy was used for all the datasets. Firstly, the raw data were processed by MotionCor2, which were aligned and averaged into motion-corrected summed images. After that, the defocus value for each micrograph was determined using Gctf. Next micrographs of high quality were selected for further processing. Then particles were picked and extracted for two-dimensional alignment. Out of these, the well-defined particles were selected for initial model reconstruction. The initial model was used as reference for 3D classification. After the refinement and post-processing, the overall resolution of complex was up to 3.9 Å based on the gold-standard Fourier shell correlation (threshold = 0.143). The quality of the local resolution was evaluated by ResMap.

**Model building and refinement**

The atom models of the complexes were generated by first fitting the chains of native apo RABV-G trimer (PDB: 8A1E) into the obtained cryo-EM densities by Chimera. The model of CTB011 and CTB012 Fab were predicted by AlphaFold2. Then the structure was manually adjusted and corrected according to the protein sequences and cryo-EM densities in Coot, and finally, real-space refinement was performed by Phenix. Details of the refinement statistics of the complexes are summarized in Table S2.

**RABV sequences analysis**

The occurrence of different amino acid identities at antigenic site of CTB011 and CTB012 were analyzed by downloading totally 1136 RABV glycoprotein sequences and 1216 lyssavirus glycoprotein sequences present in National Center for Biotechnology Information (NCBI). These sequences were aligned using MAFFT to perform the amino acid distribution analysis. All algorithms are written in Python. A Multiple sequence alignment of amino acid sequences was performed on the full-length sequences using Clustal omega. Phylogenetic analysis of these sequences was then undertaken using the maximum likelihood method available in the PhyML package.

**Biolayer Interferometry (BLI)**

Biolayer interferometry (BLI) experiments were conducted using an Octet Red96 instrument (Pall Fortebio). The running buffers used were PBS at pH 7.5 or pH 5.5, supplemented with 0.02% (v/v) Tween-20 and 0.1% (w/v) bovine serum albumin (BSA). Recombinant antibodies, CTB011 or CTB012, were immobilized on anti-human IgG Fc capture (AHC) sensors. Following immobilization, the sensors were washed with buffer, and recombinant RABV-G at pH 7.5 was introduced to bind the antibodies. After binding, the sensors were washed with running buffer at pH 5.6 for 120 seconds. To assess the conformation of RABV-G, Fab fragments that specifically bound to the prefusion form of G were incubated for over 10 minutes in the pH 5.6 running buffer before testing.

**Neutralization Assay**

Neutralization assay used by Canadian Food Inspection Agency (CFIA): Serial four-fold dilutions of SYN023 (starting concentration of 0.005 mg/mL), HRIG at 2 IU/mL (Imogam, Sanofi Pasteur), negative control mAb, raised against canine adenovirus, in hybridoma supernatant (CFIA), and the WHO 2nd International Standard for Anti-Rabies Immunoglobulin (WHO Std) at 1 IU/mL (National Institute of Biological Standards and Controls, Potter’s Bar, UK) were prepared directly in 96-well plates (Falcon) using MEM-10-5 and a final volume of 60 µL/well, with four replicate wells per dilution. An equal volume of virus suspension at approximately 1.7×10^3^ TCID50/mL was added to each well and the plate was incubated for 60 min at 37°C, after which 100 µL of MNA cell suspension (8×10^4^ cells/mL) was added to each well. After 7 d incubation, the monolayers were fixed with cold acetone (75% v/v) and virus was detected with a FITC-conjugated anti-rabies ribonucleoprotein polyclonal goat antibody (CFIA). The antibody titer was calculated using the Spearman-Kärber formula (50% endpoint). A titer of 2 is achieved if there is no neutralization observed, i.e., if all wells contained virus after 7 days. Titers were also expressed in IU/mL by normalization against the titer obtained for the WHO standard and for HRIG, provided a titer of at least 16 was obtained for these antibodies. For a test to be considered valid the negative control mAb must have a titer <8 and the virus titer must be between 32 and 316. Each virus was tested in three independent neutralization assays and results expressed as the geometric mean of the replicate titers.

Neutralization assay used by Centers for Disease Control and Prevention (CDC): Eight 5-fold serial dilutions of SYN023, HyperRAB (HRIG) and SRIG were incubated with RABV variants in 8-well tissue culture chamber slides for 90 min at 37°C. SYN023 were tested at two different concentrations, 5 μg/ml or 1 mg/ml depending on the RABV variants, along with the buffer dilutant as a negative control. For every run, RABV CVS-11 virus was included as a standard positive control to demonstrate neutralization with mAbs, HyperRAB (HRIG) and SRIG. 200μL of MNA cells (5×10^5^ cells/ml) were then added to every well containing the antibody-virus mixture, which is comprised of 50 μL of serum and 100 μL of the RABV and incubated for an additional 20 hours at 37°C with 0.5% CO_2_. Slides were then washed, fixed with acetone and stained with anti-rabies FITC (fluorescein isothiocyanate) immunoglobulin (Fujirebio Diagnostics, Inc) containing Evans blue (0.5% in PBS). Evans blue is a counterstain that provides a red background fluorescence to improve contrast. Twenty distinct microscopic fields per well were examined using a fluorescence microscope at ×200 magnification to score the virus-infected cells (foci). From the number of positive fields per well, the rabies VNA titers are mathematically calculated using the Reed-Muench calculation. The endpoint neutralization titer for CVS-11 was converted to international units (IU)/ml values by calibration against the endpoint neutralization titer of the U.S. Standard Rabies Immune Globulin (SRIG) (obtained from Food and Drug Administration, U.S.), which was measured in the same assay at 2.0 IU/ml. The experiments were performed in triplicates.

**Table S1. Cryo-EM data collection, refinement and validation statistics of TSWV-L structures.**

|  | **RABV-G-CTB011-CTB012 complex** |
| --- | --- |
| **Magnification** | 130,000 |
| **Voltage (kV)** | 300 |
| **Electron exposure (e–/Å^2^)** | 60 |
| **Defocus range (μm)** | -1.2 ~-1.8 |
| **Pixel size (Å)** | 1.04 |
| **Symmetry imposed** | C1 |
| **Initial particle images** | 845,312 |
| **Final particle images** | 359,068 |
| **Map resolution (Å)** | 3.9 |
| **Map resolution range (Å)** | 4.0-60 |
| **Initial model used (PDB code)** | none |
| **Model resolution (Å)**  **FSC threshold** | 3.2  0.143 |
| **Map sharpening *B* factor (Å^2^)** | -200 |
| **Model composition**  **Non-hydrogen atoms**  **Protein residues**  **Ligands** | 12,717  1,580  0 |
| ***B* factors (Å^2^)**  **Protein**  **Ligand**  **Nucleotide** | 129.97  0.00  0.00 |
| **R.m.s. deviations**  **Bond lengths (Å)**  **Bond angles (°)** | 0.002  0.917 |
| **Validation**  **MolProbity score**  **Clashscore**  **Poor rotamers (%)** | 2.26  18.45  0.00 |
| **Ramachandran plot**  **Favored (%)**  **Allowed (%)**  **Disallowed (%)** | 91.84  7.78  0.39 |

**Table S2. Residues of RABV G interacting with CTB011 Fab at the binding interface (d < 4.5 Å)**

| **RABV G** | | **CTB011 Fab** | | |
| --- | --- | --- | --- | --- |
| **Residues** |  |  |  | **Residues** |
| R352 |  |  |  | Y94(Light Chain) |
| T353 |  |  |  | S59(Heavy Chain) |
| N355 |  |  |  | D50(Heavy Chain) |
|  |  |  |  | S59(Heavy Chain) |
| E356 |  |  |  | S93(Light Chain) |
|  |  |  |  | Y94(Light Chain) |
| P359 |  |  |  | I33(Heavy Chain) |
|  |  |  |  | Q99(Heavy Chain) |
| S360 |  |  |  | Y52(Heavy Chain) |
|  |  |  |  | D102(Heavy Chain) |
| K361 |  |  |  | Y52(Heavy Chain) |
|  |  |  |  | D31(Heavy Chain) |
|  |  |  |  | Y32(Heavy Chain) |
| R365 |  |  |  | N104(Heavy Chain) |
| G368 |  |  |  | N104(Heavy Chain) |
| R369 |  |  |  | N104(Heavy Chain) |
| D387 |  |  |  | Y54(Heavy Chain) |
| G388 |  |  |  | Y54(Heavy Chain) |
|  |  |  |  | Y55(Heavy Chain) |
| N389 |  |  |  | Y54(Heavy Chain) |
|  |  |  |  | Y55(Heavy Chain) |
| V390 |  |  |  | Y55(Heavy Chain) |
| M395 |  |  |  | Y55(Heavy Chain) |
| L399 |  |  |  | Y55(Heavy Chain) |

**Table S3. Residues of RABV G interacting with CTB012 Fab at the binding interface (d < 4.5 Å)**

| **RABV G** | | **CTB012 Fab** | | |
| --- | --- | --- | --- | --- |
| **Residues** |  |  |  | **Residues** |
| L47 |  |  |  | Y57(Heavy Chain) |
| V48 |  |  |  | T56(Heavy Chain) |
|  |  |  |  | Y57(Heavy Chain) |
| V49 |  |  |  | T56(Heavy Chain) |
| E50 |  |  |  | S52(Heavy Chain) |
|  |  |  |  | S53(Heavy Chain) |
|  |  |  |  | G54(Heavy Chain) |
|  |  |  |  | T56(Heavy Chain) |
|  |  |  |  | Y57(Heavy Chain) |
| E52 |  |  |  | T28(Heavy Chain) |
|  |  |  |  | S30(Heavy Chain) |
|  |  |  |  | G31(Heavy Chain) |
| S284 |  |  |  | R102(Heavy Chain) |
| D285 |  |  |  | R102(Heavy Chain) |
| E286 |  |  |  | R101(Heavy Chain) |
|  |  |  |  | R102(Heavy Chain) |
|  |  |  |  | Y104(Heavy Chain) |
| I287 |  |  |  | Y104(Heavy Chain) |
| E288 |  |  |  | R99(Heavy Chain) |
|  |  |  |  | Y104(Heavy Chain) |
|  |  |  |  | Y105(Heavy Chain) |
| H289 |  |  |  | Y104(Heavy Chain) |
|  |  |  |  | Y105(Heavy Chain) |
|  |  |  |  | D37(Light Chain) |
| L290 |  |  |  | L99(Light Chain) |
|  |  |  |  | F101(Light Chain) |
| V291 |  |  |  | Y57(Heavy Chain) |

**Table S4. Contact residues and neutralization efficacy of SYN023 and HRIG against lyssavirus variants**

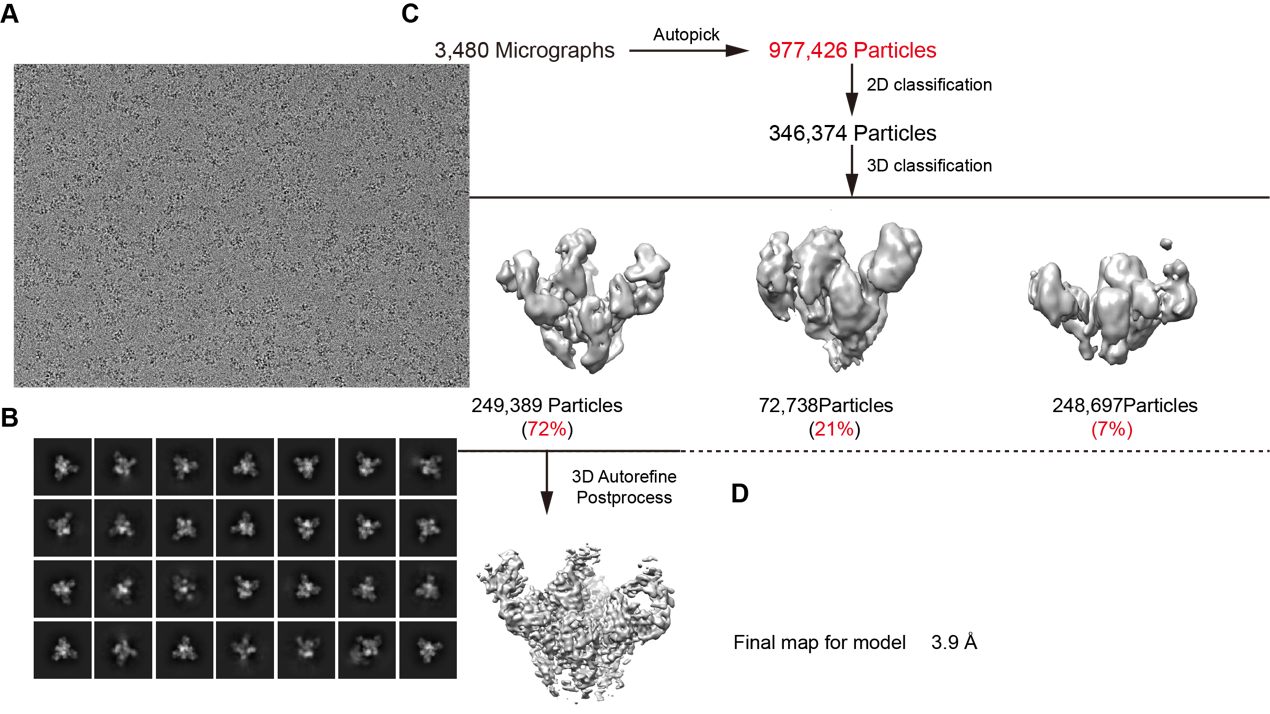


**Fig S1. Flowcharts for RABV G-trimer in complex with CTB011 and CTB012 Fab.** Electron micrograph (**a**), 2D class average images (**b**) and flowchart for cryo-EM data processing (**c, d**).


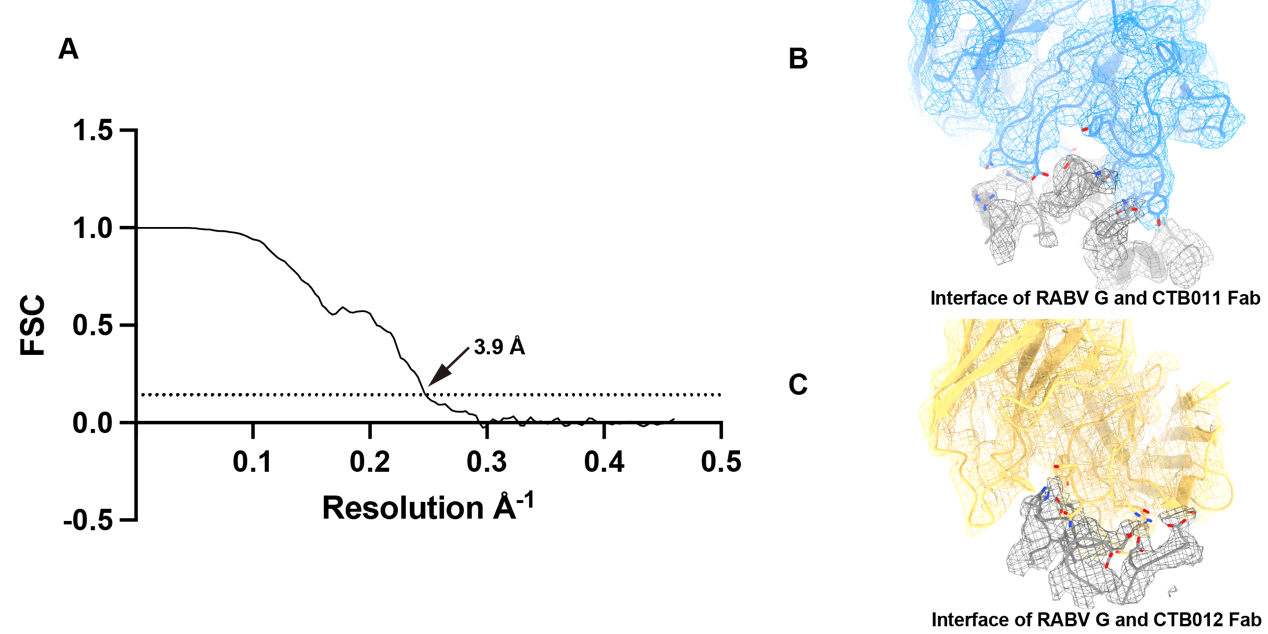


**Fig S2. Resolution of RABV G-trimer in complex with CTB011 and CTB012 Fab structure.** Fourier shell correlation (FSC) curves (**a**), density maps, and atomics models of RABV G-trimer in complex with CTB011 and CTB012 Fab (**b, c**).


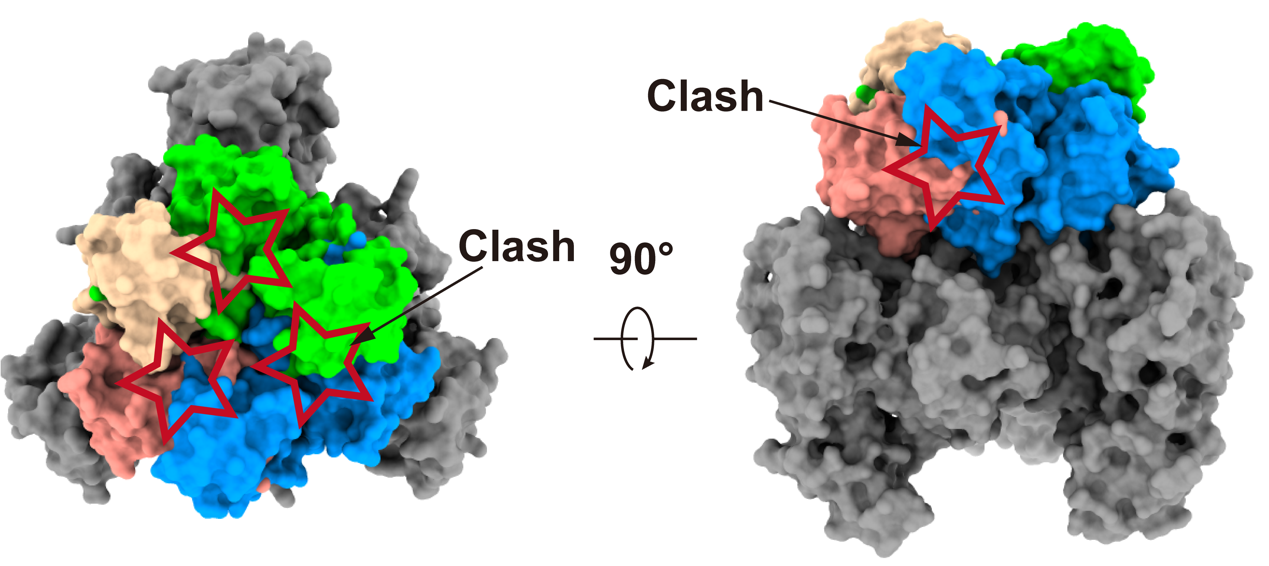


**Fig S3. The binding mode of CTB012 Fab.** Steric clashes are denoted by red stars.

**
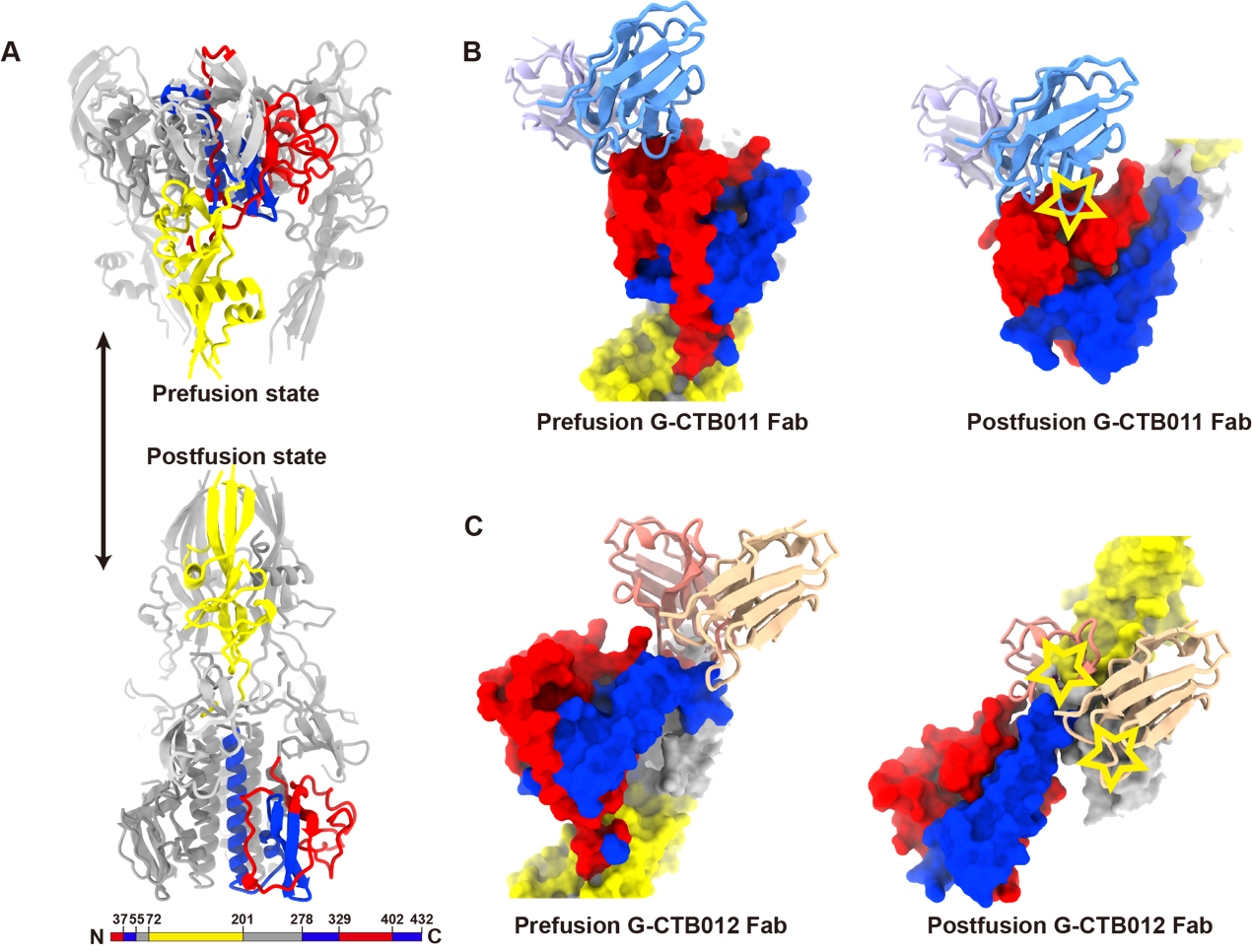
**

**Fig S4. Superposition of the predicted postfusion RABV-G model and the prefusion RABV-G structure (with CTB011/CTB012 bound to G) indicates steric incompatibility of CTB011/CTB012 Fab with the postfusion state.** (A) predicted model of the postfusion RABV-G spike. (B) and (C) Superposition of CTB011/CTB012 to the predicted postfusion and the prefusion RABV-G structure. Steric clashes are denoted by yellow stars.

**
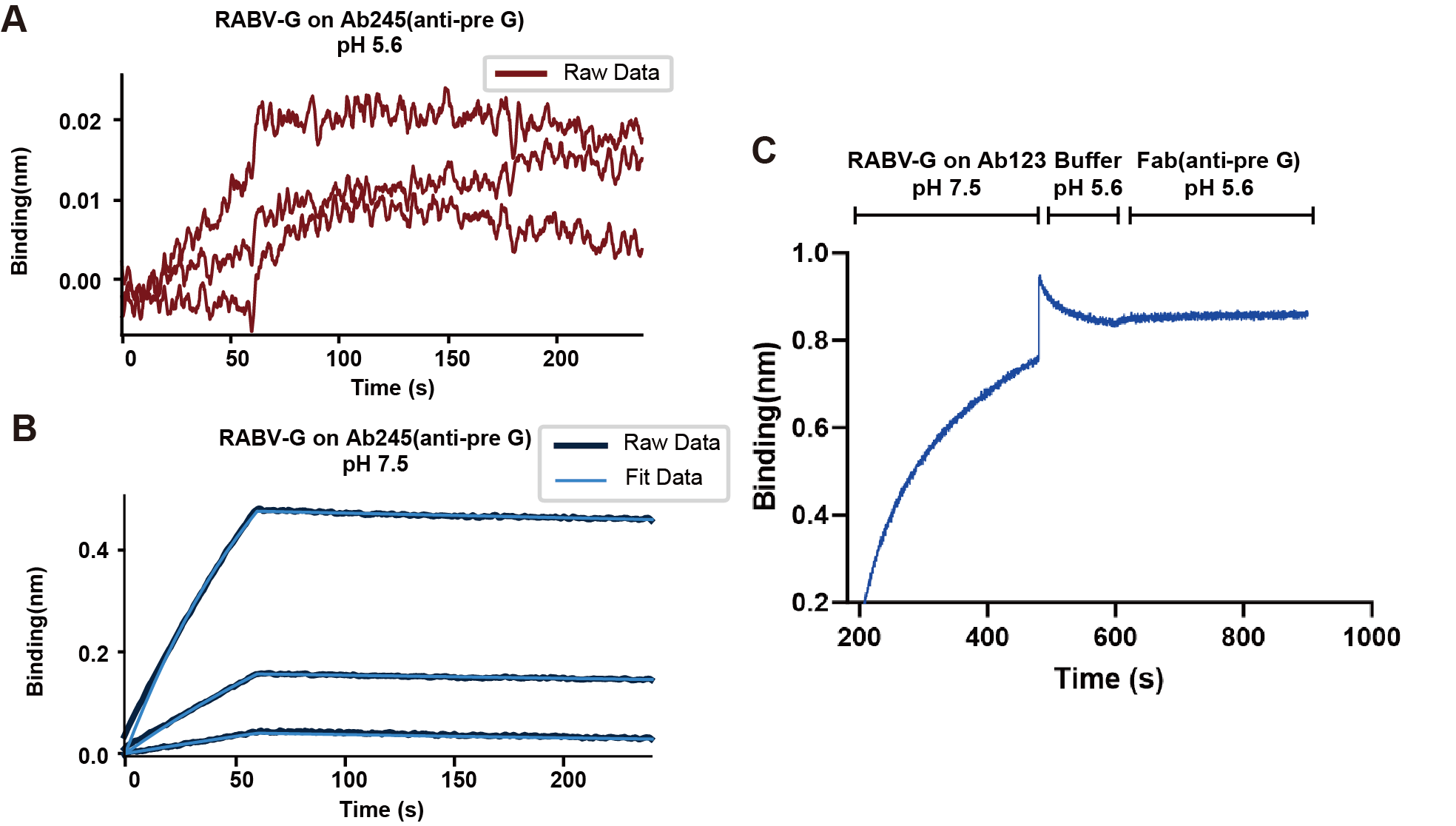
**

**Fig S5. RABV-G interactions with Fabs and antibodies under acidic and neutral pH conditions, Related to Figure 1F and 1G.** Biolayer interferometry analysis for mAb 245 interactions with RABV-G under acidic (A) and neutral (B) pH conditions. (C) The “sandwich-configuration” BLI assay, captured by a non-neutralizing control antibody (mAb123).
